# Loss of macroH2A1 decreases mitochondrial metabolism and reduces the aggressiveness of uveal melanoma cells

**DOI:** 10.18632/aging.103241

**Published:** 2020-05-12

**Authors:** Sebastiano Giallongo, Michelino Di Rosa, Rosario Caltabiano, Lucia Longhitano, Michele Reibaldi, Alfio Distefano, Oriana Lo Re, Angela Maria Amorini, Lidia Puzzo, Lucia Salvatorelli, Stefano Palmucci, Daniele Tibullo, Andrea Russo, Antonio Longo, Giacomo Lazzarino, Giovanni Li Volti, Manlio Vinciguerra

**Affiliations:** 1 Department of Biomedical and Biotechnological Sciences, University of Catania, Catania, Italy; 2 Center for Translational Medicine (CTM), International Clinical Research Center (FNUSA-ICRC), St Anne's University Hospital, Brno, Czech Republic; 3 Department of Biology, Faculty of Medicine, Masaryk University, Brno, Czech Republic; 4 Department G.F. Ingrassia, Section of Anatomic Pathology, University of Catania, Catania, Italy; 5 Department of Ophthalmology, University of Catania, Catania, Italy; 6 UniCamillus-Saint Camillus International University of Health Sciences, Rome, Italy; 7 EuroMediterranean Institute of Science and Technology, Palermo, Italy

**Keywords:** macroH2A1, histones, uveal melanoma, metabolism, epigenetics

## Abstract

Uveal melanoma (UM) is the most common primary intraocular tumour in adults. The most accurate prognostic factor of UM is classification by gene expression profiling. Currently, the role of epigenetics is much less defined compared to genetic mechanisms. We recently showed a strong prognostic role of the expression levels of histone variant macroH2A1 in UM patients. Here, we assessed the mechanistic effects of macroH2A1 on UM progression.

UM cell lines were stably knocked down (KD) for macroH2A1, and proliferation and colony formation capacity were evaluated. Mitochondrial function was assayed through qPCR and HPLC analyses. Correlation between mitochondrial gene expression and cancer aggressiveness was studied using a bioinformatics approach.

MacroH2A1 loss significantly attenuated UM cells proliferation and aggressiveness. Furthermore, genes involved in oxidative phosphorylation displayed a decreased expression in KD cells. Consistently, macroH2A1 loss resulted also in a significant decrease of mitochondrial transcription factor A (TFAM) expression, suggesting impaired mitochondrial replication. Bioinformatics analyses uncovered that the expression of genes involved in mitochondrial metabolism correlates with macroH2A1 and with cancer aggressiveness in UM patients. Altogether, our results suggest that macroH2A1 controls UM cells progression and it may represent a molecular target to develop new pharmacological strategies for UM treatment.

## INTRODUCTION

Uveal melanoma (UM) is the most common primary intraocular tumour in adults [1, 2]. Metastasis is a frequent occurrence in UM with a 5 years survival of ~15%. It is estimated that 40-50% of UM patients will die of metastatic disease, even with early diagnosis and proper treatment [3]. By far the most common site of UM metastasis is the liver, reported in ~87% of metastasis cases [4]. Although both uveal and cutaneous melanomas arise from melanocytes, UM is biologically and genetically distinct from the more common cutaneous melanoma [5]. In particular, UM lack mutations in BRAF, NRAS, or KIT, unlike cutaneous melanoma and it is characterized by activating mutations in the GPCR alpha subunits GNAQ or GNA11 [6]. Moreover, inactivating somatic mutations in the gene encoding BRCA-1 associated protein 1 (BAP1) have been observed in ~84% of metastasizing UM [7]. The frequency of BAP1 mutations in metastatic UM suggests that targeting the BAP1 pathway could be a valuable therapeutic approach. BAP1 is an enzyme that mediates epigenetic modifications like deubiquitination of histone H2A and host cell factor 1 (HCF-1) [8–10]. Epigenetic mechanisms controlling gene expression have long been known to have a role in cancer development [11]. In UM these include DNA methylation at CpG islands in promoters leading to decrease expression of p16/INK4a tumour suppressor protein [12]. However, compared with the genetic mechanisms, the role of epigenetics in UM carcinogenesis is poorly defined [13–16].

Histone variants confer unique biological functions to chromatin [17–19]. The H2A family is the most diverse and includes vertebrate-specific macroH2A1 (with splice variants mH2A1.1 and 1.2) and macroH2A2 [20–22], which are generally associated with transcriptionally repressed chromatin [23, 24]. However, macroH2A histones are widely distributed throughout chromatin, but may also exert positive effects [20–22, 25, 26]. Recent studies have examined the expression of macroH2A1 in solid tumours and its correlation with clinical pathological features, including cutaneous melanoma [27–31]. MacroH2A1 appears to act as tumour suppressor or as an oncogene depending on the type of cancer and on the degree of stemness [21, 27, 28, 30]. Contrary to cutaneous melanoma [27], we demonstrated immunohistochemically that macroH2A1 expression is higher in metastatic UM than in not metastatic UM [32]. However, the role of macroH2A1 in UM development and progression is unknown. A better understanding of the epigenetic processes leading to UM progression, metastasis and mortality, is needed to identify new prognostic markers for the early diagnosis or response to treatment. The aim of the present study was to assess the role of histone macroH2A1 in UM progression and related metabolic pathways involved in cell proliferation and metastasis, using cell models and biopsies from UM patients.

## RESULTS

### MacroH2A1 silencing reduces cell proliferation and migration

**Figure 1:**
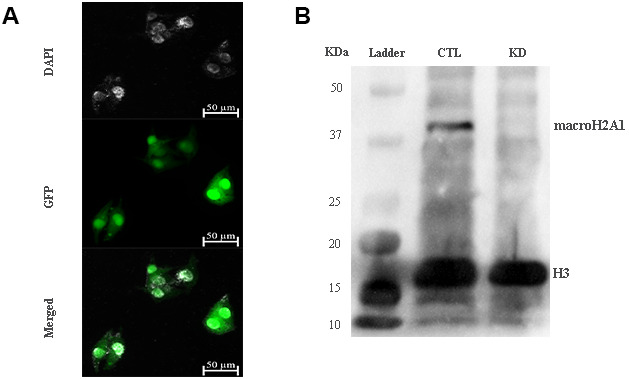
**macroH2A1 silencing (knock-down, KD) in UM 92.1 cells.** (**A**) Cells were infected with a lentivirus bearing a bicistronic construct expressing GFP and a macroH2A1-silencing shRNA. Control cells (CTL) were infected with lentivirus bearing a bicistronic construct expressing GFP and a scramble shRNA (*data not shown*). (**B**) Western blot analysis showed a significant reduction of macroH2A1 in transfected cells.

**Figure 2:**
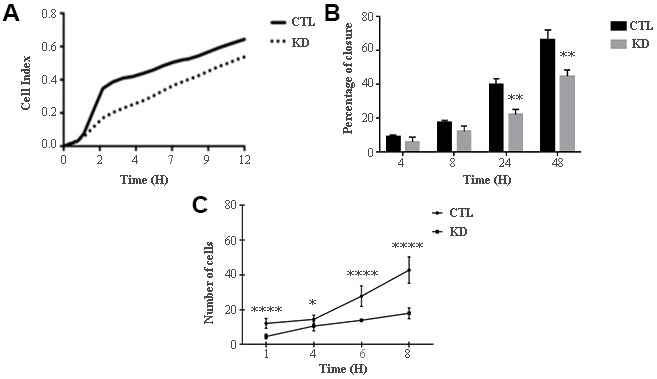
**Proliferation and migration of UM 92.1 cells KD for macroH2A1.** (**A**) Real time cell proliferation monitored by xCELLigence system. Cell index values were normalized at the time zero in order to obtain a normalized cell index. Each line is expressing the average of four different experiments. (**B**) Cell proliferation assay. Values are presented as percentage of the open wound following 4, 8, 24 and 48 hours (wound at time 0 was assumed as 100% and used as control). Values are expressed as the mean ± SEM of three different experiments. (*p* < 0.0001 vs control). (**C**) The migration assay. Values are presented as number of migrating cells following 1, 4, 6 and 8 hours (wound at time 0 was assumed without migrating cells and used as control). Values are expressed as the mean ± SEM of three independent experiments. (*p* < 0.0001 vs control).

**Figure 3:**
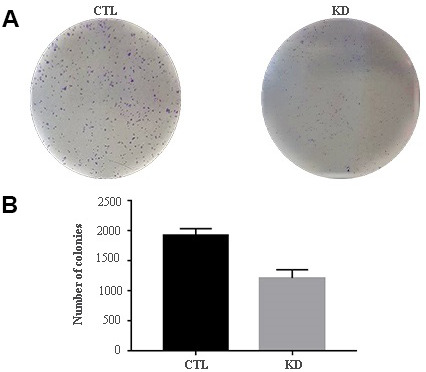
**Colony formation capacity of UM 92.1 cells KD for macroH2A1.** UM 92.1 cells were silenced for macroH2A1 expression as in Figure 1. (**A**) Images are representative of three separate experiments. (**B**) The number of colonies was manually counted and presented as the mean ± SEM of three independent experiments. (*p < 0.01 vs control).

The role of macroH2A1 was investigated by lentiviral mediated silencing in UM 92.1 cells. Control cells (CTL) were infected with a bicistronic construct expressing green fluorescent protein (GFP) and a scramble shRNA, while silencing of macroH2A1 was achieved through lentiviral infection of a bicistronic construct containing shRNA against macroH2A1 and a GFP cassette [29, 33] (knock-down, KD, Figure 1A). Lentiviral-mediated shRNA against macroH2A1 was confirmed by immunoblotting (Figure 1B). Our group already showed that loss of macroH2A1 leads to increased stemness and decreased proliferation in liver cancer cells [29, 33]. Here, we generated cell growth curves for control and KD UM 92.1 cells using xCELLigence. Cells were seeded in wells carrying a gold electrode that measures electric impedance. The latter is related to cell density on the chip and is converted automatically into cell index by the device. Evaluation of cell index per each time point provides a direct quantification of cell proliferation [34]. XCELLigence analysis showed that macroH2A1 loss leads to a significant decrease of proliferation rate when compared to their control (p < 0.01) (Figure 2A). Interestingly, wound healing assay showed that silencing of macroH2A1 decreases wound closure ability of 92.1 UM cells (Figure 2B, Supplemental Figure 1). The difference became significant (p < 0.01) 24 hours after scrape introduction: at this time point the control and silenced cells differ for ~50% in wound closure (Figure 2B). Moreover, knockdown of macroH2A1 resulted into a decrease in migration in serum starved 92.1 UM cells (Figure 2C, Supplemental Figure 2). The difference in migration became highly significant (p < 0.0001) 6 hours after the introduction of the scrape. At this time point the number of KD cells migrated are a half compared with CTL cells (Figure 2C). These findings were further confirmed by clonogenic assay. Upon macroH2A1 knockdown 92.1 UM cells decreased their colony formation capacity (Figure 3A). Moreover, the plate efficiency (% of cells inoculated at a low density that gave rise to colonies) of KD cells was significantly decreased compared with CTL cells (24.3 ± 4.81 *versus* 38.42 ± 4.04). Therefore, macroH2A1 silencing in UM cells significantly hampers their ability to proliferate and to migrate.

### MacroH2A1 silencing decreases mitochondrial metabolism in UM cells

**Figure 4:**
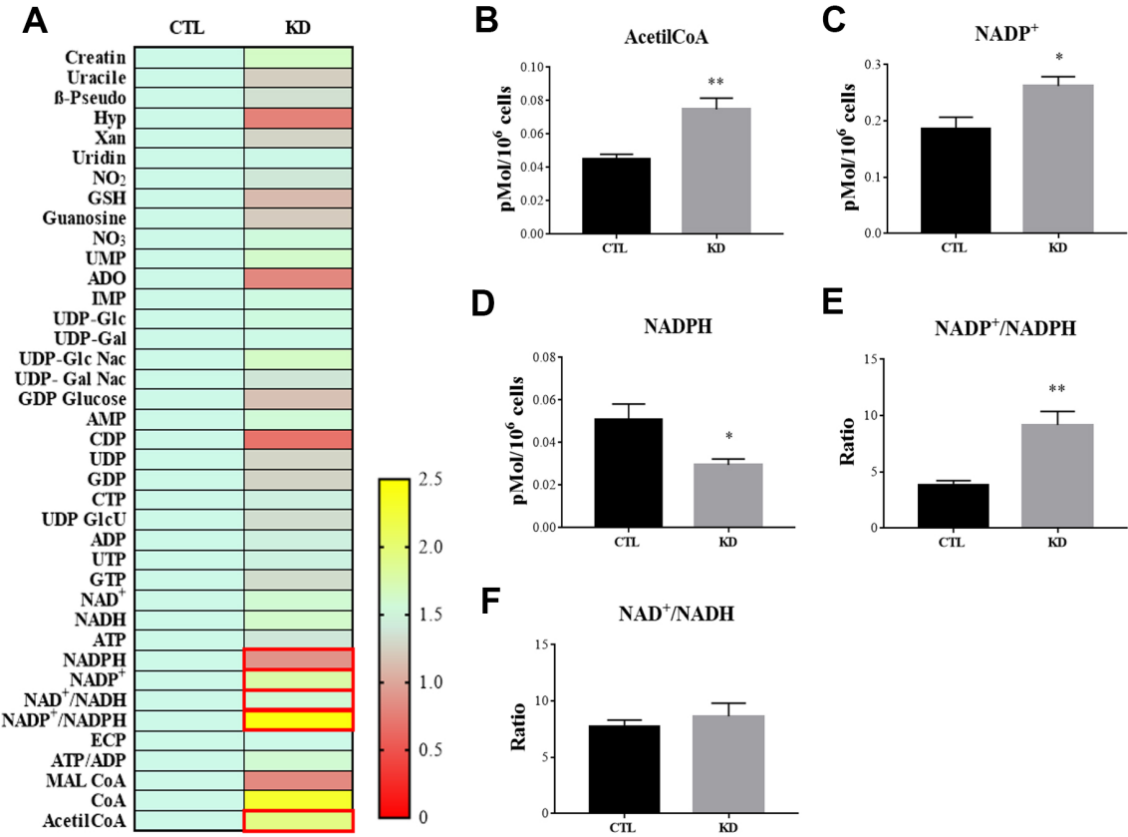
**HPLC analysis of metabolites in CTL and macroH2A1 KD UM 92.1 cells.** (**A**) Heatmap representing the levels of major classes of metabolites detected by HPLC. (**B**) Acetyl-co-A levels; (**C**) NADP^+^ levels; (**D**) NADPH levels; (**E**) NADP^+^/NADPH levels; (**F**) NAD^+^/NADH levels. Results are presented as the mean ± SEM of four independent experiments. (*p < 0.01; *** p < 0.001 vs control).

**Figure 5:**
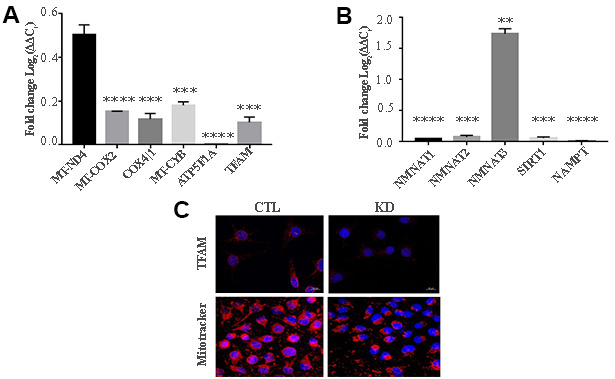
**KD for macroH2A1 reprograms energy metabolism in UM 92.1 cells.** (**A**) mRNA levels MT-ND4, MT-CO2, COX4|1, MT-CYB, ATP5F1A and TFAM were assessed by qPCR, and normalized to GAPDH levels. Values are presented as mRNA relative levels and they are expressed as the mean ± SEM of three different experiments. (p < 0.0001 vs control). (**B**) mRNA levels NMNAT1, NMNAT2, SIRT1 and NAMPT. Values are presented as mRNA relative levels and they are expressed as the mean ± SEM of three different experiments. (**p<0.01****p < 0.0001 vs control). (**C**) Representative immunocytochemical images showing staining for TFAM (upper panels) and MitoTracker (lower panels) in CTL and in UM 92.1 cells KD for macroH2A1.

MacroH2A1 KD is able to increase lipid synthesis and to activate glycolytic pathways and in particular the pentose phosphate pathway (PPP) in HCC cells, rewiring energy metabolism to the needs of a cancer stem cell (CSC)-like state [29, 33, 35]. To study the role of macroH2A1 in the energy metabolism of UM cells, we next analyzed the endogenous metabolic profiles of control and macroH2A1 KD UM 92.1 cells. Metabolic profiling relies on the ability to determine changes in the total complement of metabolites in cells. In Figure 4A we report a heatmap representing all the changes in the levels of metabolites tested by high-performance liquid chromatography (HPLC), which allows the separation and quantification of most metabolites from glycolysis and the Krebs cycle including the high energy phosphates. Consistent with previous observations in HCC cells [33], macroH2A1 silencing resulted in a significant increase of acetyl-CoA (Figure 4B) and NADP^+^ (Figure 4C) content accompanied by a significant decrease of NADPH (Figure 4D), thus suggesting a switch to reductive biosynthesis and to lipid synthesis in KD cells. Consistent with this, the NADP^+^/NADPH ratio is increased in UM 92.1 cells knockdown for macroH2A1 (Figure 4E), while the ratio NAD^+^/NADH showed a trend to be higher upon macroH2A1 silencing (Figure 4F). As consequence, the impaired lipid biosynthesis reflected into a decreased efficiency of the pentose phosphate pathway (PPP) as also supported by the decreased trend of nucleic acid precursor CDP (cytosine diphosphate) and Hyp (hypoxanthine) (Figure 4A). MacroH2A1.1 isoform has been shown to boost mitochondrial respiration when overexpressed in muscle cells [36]. Conversely, we hypothesized that macroH2A1 KD in UM 92.1 cells might hamper the activity of mitochondria. We thus analyzed expression of genes involved in oxidative phosphorylation: the expression of MT-ND4, MT-CO2, COX4|1, MT-CYB, ATP5F1A and TFAM mRNAs were significantly decreased in KD UM cells compared to their controls (p < 0.001) (Figure 5A). The maintenance of an optimal NAD^+^/NADH ratio is essential for mitochondrial function [37]; UM 92.1 cells KD for macroH2A1 showed also a significant (p < 0.001) decrease in the mRNA levels of NMNAT1, NMNAT2, SIRT1 and NAMPT, key enzymes implicated in NAD^+^ turnover [38] (Figure 5B). In contrast, the mRNA levels of NMNAT3 were increased of >1.5 in UM cells KD for macroH2A1 (Figure 5B). Interestingly, T-Fam transcript was also found significantly downregulated in UM 92.1 cells KD for macroH2A1 compared to CTL cells (p < 0.001) (Figure 5B). The TFAM gene encodes for the mitochondrial transcription factor A (TFAM), essential for replication and packaging of mitochondrial DNA into nucleoids, as well as critical for mitochondrial biogenesis [39]. Consistently, imaging for viable mitochondria co-stained with MitoTracker and TFAM antibody showed an impaired mitochondrial status in 92.1 UM cells deficient for macroH2A1 (Figure 5C).

### MacroH2A1 gene expression (H2AFY) regulates the expression of genes involved in mitochondrial metabolism in UM patients

**Figure 6:**
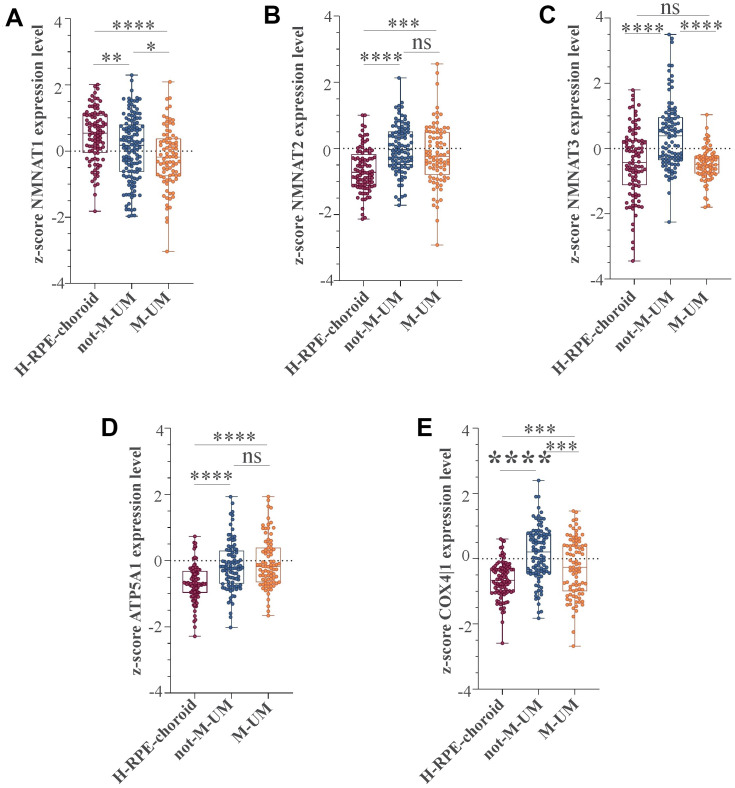
**NMNAT1, NMAT2, NMNAT3, ATP5F1A, and COX4|1 mRNA expression levels in UM patients.** Expression levels analysis of (**A**) NMNAT1, (**B**) NMAT2, (**C**) NMNAT3, (**D**) ATP5F1A, and (**E**) COX4|1 in 96 healthy control subjects (H-RPE-choroid), 88 metastatic (M-UM) and 102 non-metastatic (not-M-UM) UM patients. Data are expressed as z-score intensity expression levels and presented as vertical scatter dot plots. P values <0.05 were considered to be statistically significant (*p<0.05; **p<0.005;***p<0.0005; ****p<0.00005).

**Figure 7:**
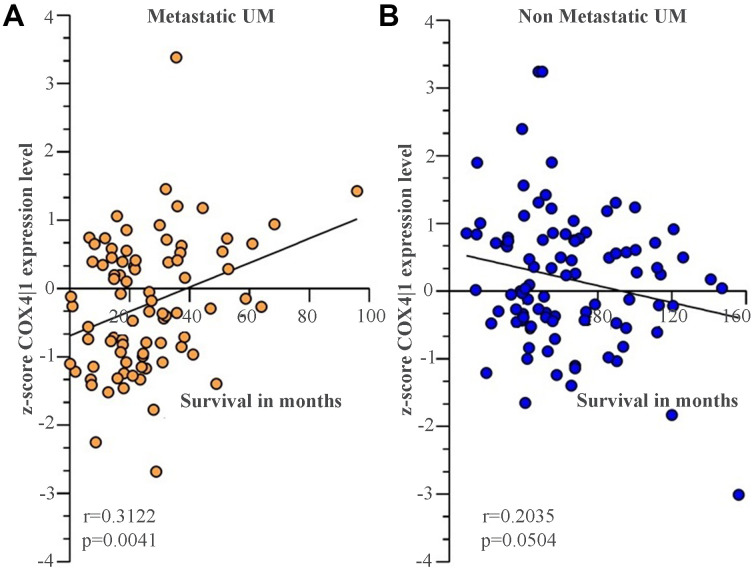
**COX4|1 mRNA expression levels correlate with the surviving rate in UM metastatic patients.** Correlation analysis of COX4|1 with surviving rate in (**A**) 88 metastatic (M-UM) and 102 (**B**) non-metastatic (not-M-UM) UM patients. Data are expressed as z-score intensity expression levels and presented as vertical scatter dot plots. Correlations were determined using Pearson’s ρ correlation. P values <0.05 were considered to be statistically significant (*p<0.05; **p<0.005;***p<0.0005; ****p<0.00005).

We recently conducted a retrospective study on macroH2A1 immunohistochemical expression in 55 UM patients, demonstrating a positive immunohistochemical correlation between macroH2A1 levels and UM aggressiveness [32]. Here we sought to analyse larger cohort to examine the correlation between the genes involved in mitochondrial metabolism and UM aggressiveness. To this aim, we took into account a total of 190 samples of patients with UM and 96 retinal pigment epithelium (RPE)-choroid of healthy control subjects, pooled from 6 different publicly available Gene Expression Omnibus (GEO) repositories [40] (GSE44295, GSE22138, GSE27831, GSE84976, GSE51880, GSE73652, GSE29801) (Table 1). From all the selected datasets we were able to obtain data regarding sex, age, presence of metastases, and survival rate. The GSE73652 dataset did not present information regarding sex. We divided the UM patients according to the presence or absence of metastases. Two groups of metastatic (88) and non-metastatic (102) patients were compared with each other and with the control group composed of healthy subjects (96), for the expression of the same panel of genes analyzed in UM 92.1 cells KD for macroH2A1 and involved in mitochondrial respiration and NAD^+^ metabolism (Figure 5) (MT-ND4, MT-CO2, COX4|1, MT-CYB, ATP5A1 and TFAM, NMNAT1, NMNAT2, NMNAT3 SIRT1 and NAMPT). Our analysis highlighted that for NMNAT2, NMNAT3, COX4|1 and ATP5A1 expression levels were significantly increased in UM (metastatic/non metastatic [32]) patients compared to healthy controls (Figure 6A–6E). No differences were observed for MT-ND4, MT-CO2, MT-CYB, TFAM, SIRT1 and NAMPT across the three categories (*data not shown*). Interestingly, when comparing metastatic versus non metastatic UM patients, we observed a downregulation of the NMNAT1, NMNAT3 and COX4|1, but not of NMNAT2 and ATP5A1, mRNA levels (Figure 6A–6E). This is consistent and mirrors our data in macroH2A1 KD cells, less proliferative and aggressive, where we observed a general downregulation of the mRNA levels of the enzymes involved in NAD^+^ metabolism (Figure 5). Next, we sought to ascertain whether the expression levels of the selected genes were significantly correlated with the UM patient’s survival rate. We found that only the COX4|1expression levels were significantly positively correlated with the survival rate of metastatic patients (r=0.3122, p=0.0041) (Table 2A, Figure 7A). A nearly significant negative correlation between COX4|1 and survival was observed with non-metastatic patients (r=-0.2035, p=0.0504) (Table 2B, Figure 7B). Altogether these data demonstrate the importance of mitochondrial metabolism, as assessed by gene expression, in UM occurrence and aggressiveness.

**Table 1:** List of GEO datasets selected.

**GSE**	**GPL**	**Disease**	**Sample**	**Male**	**Female**	**Metastasis**	**No Metastasis**
**44295**	6883	UM	57	32	25	24	33
**22138**	570	UM	63	39	24	35	28
**27831**	570	UM	29	17	12	11	18
**84976**	10558	UM	28	ns	ns	13	15
**73652**	10558	UM	13	ns	ns	5	8
**29801**	4133	UM	96	58	38	Not affected	Not affected

**Table 2:** Pearson correlation analyses between gene expression and survival in UM patients.

**A**					
**Metastatic**	**surviving months vs. NMNAT1**	**surviving months vs. NMNAT2**	**surviving months vs. NMNAT3**	**surviving months vs. ATP5A1**	**surviving months vs. COX4I1**
**Pearson r**	-0.1516	0.1291	0.1098	0.05273	0.3122
**95% confidence interval**	-0,3556 to 0,06628	-0,08907 to 0,3354	-0,1085 to 0,3179	-0,1648 to 0,2654	0,1035 to 0,4946
**R squared**	0.02297	0.01667	0.01205	0.00278	0.09748
**P (two-tailed)**	0.1714	0.2448	0.3233	0.6359	0.0041
**P value summary**	ns	ns	ns	ns	**
**Significant (alpha = 0.05)**	No	No	No	No	Yes
**Number of XY pairs**	83	83	83	83	83
**B**					
**Non Metastatic**	**surviving months vs. NMNAT1**	**surviving months vs. NMNAT2**	**surviving months vs. NMNAT3**	**surviving months vs. ATP5A1**	**surviving months vs. COX4I1**
**Pearson r**	0.1271	0.07544	0.04138	0.05706	-0.2035
**95% confidence interval**	-0,07869 to 0,3224	-0,1303 to 0,2749	-0,1637 to 0,2430	-0,1484 to 0,2578	-0,3910 to 0,0002075
**R squared**	0.01614	0.005691	0.001712	0.003255	0.04142
**P (two-tailed)**	0.2249	0.4723	0.6937	0.587	0.0504
**P value summary**	ns	ns	ns	ns	ns
**Significant? (alpha = 0.05)**	No	No	No	No	No
**Number of XY pairs**	93	93	93	93	93

## DISCUSSION

Epigenetic changes cooperate actively with genetic alterations to drive the cancer phenotype. These changes involve DNA methylation, histone modifiers and readers, chromatin remodelers, ncRNA and other physical components of chromatin such as the histone variants [41]. During carcinogenesis, the result of the interplay between oncogenes and tumor suppressor genes can sometime code for histone variants [22, 42]. Others and we have shown that H2A histone variant macroH2A1 can act as oncogene or tumor suppressor depending on the blood or solid malignancy [27–31]. In the present study, we report for the first time that the loss of macroH2A1 inhibits UM cells proliferation and aggressiveness, while inducing an inhibition of mitochondrial metabolism and biogenesis through a gene expression signature that is also observed in UM patients. Therefore, consistent with previous clinical and histological studies on a an Italian national reference cohort of biopsies from UM patients, where we showed the role of macroH2A1 as prognostic marker for UM progression [32], we conclude that macroH2A1 acts as an oncoprotein in UM. At the cellular level, UM shares the same lineage – the melanocytes – with cutaneous melanoma. However, the two melanomas differ in their etiologies, clinical features, and molecular abnormalities [43]. The size of the tumor and its degree of invasion are prognostic in both entities, but the patterns of dissemination and metastasis differ: UM spreads through the blood, while cutaneous melanoma can spread through both the blood and the lymphatic system [44]. The most common site of metastasis of UM is the liver; cutaneous melanoma can instead spread to the lymph nodes, lung, brain, and soft tissue [32, 45].

The role of macroH2A1 in cutaneous melanoma has been well studied. Kapoor et al. reported 2010 that macroH2A proteins suppress progression of malignant cutaneous melanoma [27]. Loss of all macroH2A1 isoforms, positively correlated with increasing malignant phenotype of melanoma cells both in cell lines, in human tissue samples, and in animal models of cutaneous melanoma growth and metastasis; this phenotype could be restored by reintroduction of macroH2A1 [27]. The tumor suppression function of macroH2A1 in cutaneous melanoma was attributed to a large extent to the transcriptional suppression of CDK8, a known oncogene [27]. MacroH2A1 and CDK8 expression levels anti-correlate in human cutaneous melanoma patient samples [27]. Therefore, macroH2A1 functions as an oncogene in UM and as a tumor suppressor in cutaneous melanoma, highlighting the profound epigenetic differences between the two types of melanocyte-derived neoplasia. Genomic and transcriptomic approaches are required in parallel in cutaneous and UM experimental models and human biopsies to unravel the different dynamics of macroH2A1-dependent gene expression. The specific role of CDK8 in UM progression has not been investigated so far, although pan-CDK inhibition seems to be partially effective both in cutaneous melanoma and in UM [46].

Here we found that macroH2A1 loss in UM cells decrease their aggressiveness. This is supported by a distinct gene expression pattern, which is conserved between UM cultured cells and human UM datasets. The metabolite-binding macrodomain (present in macroH2A1.1 but not in macroH2A1.2) is required to sustain mitochondrial function but, interestingly is dispensable for gene regulation [36]. Resveratrol, a natural phenol, is able to inhibit tumor growth *in vitro* and in animal models of UM [47]. Consistent with our findings, an early event in resveratrol action is the direct targeting of mitochondria, which leads to a decrease in cell proliferation [47]. Similar findings were obtained with fisetin, a dietary flavonoid, and with another natural phenol, curcumin [48]. Resveratrol, fisetin and curcumin are contained in various fruits and vegetables. To date, however, there are no studies on dietary factors and incidence of UM. Half of UM patients develop liver metastases, with a median survival of > 12 months [49]. The loss of one copy of chromosome 3 (Chr3) in a primary UM, referred to as monosomy 3 (M3), is associated with metastasis and poor prognosis [50]. More than 90% of metastatic UM is M3. Consistent with our findings, M3 UM has a greater mitochondrial activity [51]. Our study identifies for the first time a correlation between the expression of COX4|1, key regulatory subunit of human cytochrome c oxidase, and UM patient survival, as observed in glioblastoma multiforme [52].

In conclusion, we suggest that strategies aiming at decreasing the expression of histone variant macroH2A1 [32], might effectively hamper the aggressiveness of UM cells, by inhibiting their mitochondrial phosphorylation. This could be a novel promising therapeutic strategy against UM [51].

## MATERIALS AND METHODS

### Cell culture

Human uveal melanoma cells (92.1) were purchased from ATCC Company (Milan, Italy). Cells were suspended in RPMI1640 culture medium containing 10% FBS, 100 U/mL penicillin, and 100 U/mL streptomycin. At 80% confluency, cells were passaged using trypsin-EDTA solution (0.05% trypsin and 0.02% EDTA).

Cell transfection was achieved using lentiviral particles [53] and carried out as previously described [28]. Cell proliferation and migration were studied using the “wound healing” assays [28]. The uncovered wound area was measured and quantified at different intervals with ImageJ 1.37v (NIH).

### Immunoblotting

Histone protein isolation was achieved using ab113476 Histone Extraction Kit (Abcam, UK). Western blot analysis was performed as previously described [54, 55]. Rabbit MacroH2A1 and H3 antibodies were from Santa Cruz Biotechnology (CA, US). Anti-rabbit HRP linked was from Cell Signaling Technology (Praha, CZ).

### Real time proliferation

xCELLigence experiments were performed using the RTCA (Real-Time Cell Analyzer) instrument (Roche Applied Science, Mannheim, Germany and ACEA Biosciences, San Diego, CA) [34]. First, the optimal seeding number was determined by cell titration and growth experiments. After seeding the optimal cell number (2500 cells/well), cells were automatically monitored every 15 min for 72h.

### qPCR

Upon mRNA extraction and cDNA reverse transcription we evaluated expression of selected genes in 92.1 cells, CTL and KD. The quantitative analysis was performed using the One-Step Real-time PCR instrument and SYBR Green PCR master mix (Life Technology, Milan, Italy) [56]. GAPDH was used for normalization. Primer sequences were: GAPDH, forward 5’-CCGCATCTTCTTTTGCGTCG-3’, reverse 3’-GACTCCGACCTTCACCTTCC-5’, MT-ND4, forward 5’- CAGCCACATAGCCCTCGTAG-3’, reverse, 3’-TCGGGGTTGAGGGATAGGAG-5’, MT-CO2, forward, 5’- GAACTATCCTGCCCGCCATC-3’, reverse, 3’-AGGGATCGTTGACCTCGTCT-5’, COX4|1, forward, 5’-GCGGTGCCATGTTCTTCATC-3’, reverse, 3’-GGGCCGTACACATAGTGCTT-5’, MT-CYB, forward 5’-TCTTGCACGAAACGGGATCA-3’, reverse 3’-TGATTGGCTTAGTGGGCGAA-5’, ATP5F1A, forward, 5’- TGTGTGTAGTCTCACGTCACC-3’, reverse, 3’- CTGCCTCATTATGGCCACTCC-5’, NMNAT1, forward 5’-CCTTGAGGGATGGCGTCAAA-3’, reverse, 3’- CTTGGCCAGCTCAAACAACC-5’, NMNAT2, forward 5’- CATGACCGAGACCACCAAGAC-3’, reverse 3’-GTCGTGGACAGGGGAGACAA-5’, SIRT1, forward, 5’- CCAAGGCCACGGATAGGTC-3’, reverse, 3’- ATTGTTCGAGGATCTGTGCC-5’, NAMPT, forward, 5’- GCTTGGGGGAAAGACCATGA-3’, reverse, 3’-GCTGACCACAGATACAGGCA-5’.

### Clonogenic assay

Colony assays performed by seeding cells in 6-wells plates at low density (5000 cells/well) and allowing growth for 9 days. Colonies were fixed, stained with crystal violet (Sigma Aldrich) and quantified using ImageJ (NIH).

### Immunofluorescence

Cells were grown directly on coverslips before immunofluorescence [57]. Briefly, after washing with PBS, cells were fixed in 4% paraformaldehyde (Sigma-Aldrich, Milan, Italy) for 20min at room temperature. Subsequently, cells were incubated with primary antibody against T-Fam we purchased from Thermo Fisher scientific (1:200), overnight at 4 °C. Cells were then washed three times in PBS for 5 min and incubated with secondary antibodies from Cell Signaling Technology. Nuclei were counterstained with DAPI (4′,6- diamidino-2phenylindole, Santa Cruz Biotechnology, CA, USA). Images were obtained using a Zeiss Axio Imager Z1 Microscope with Apotome 2 system (Zeiss, Milan, Italy).

### HPLC analysis of metabolites

Cellular packages were deproteinized to measure acid labile and easily oxidizable compounds [58]. The simultaneous separation of high-energy phosphates (ATP, ADP, AMP, GTP, GDP, GMP, IMP, UTP, UDP, UMP, CTP, CDP, CMP), Coenzyme A and its derivatives (Acetyl-CoA, Malonyl-CoA), nicotinic coenzymes (NAD^+^, NADH, NADP^+^, NADPH), reduced glutathione (GSH), malondialdehyde (MDA), nitrite and nitrate in the protein-free cell extracts, was carried out using established HPLC methods [58, 59]

### Dataset selection and analysis

The NCBI Gene Expression Omnibus (GEO) database (http://www.ncbi.nlm.nih.gov/geo/) [40] was used to select microarray datasets. Mesh terms “Uveal Melanoma” and “eyes choroid” were used to identify datasets of interest. The obtained datasets were sorted by the number of samples (High to Low) and to available clinical data. Seven datasets were selected: GSE44295, GSE22138 [60], GSE27831 [61], GSE84976 [62], GSE51880 [63], GSE73652 [64], and GSE29801 [65] (Table 1). All the selected datasets were composed of samples from UM patients, divided according to sex and the presence of metastases. The samples were homogeneous for the age. Furthermore, the samples were selected based on survival rate. The GSE29801 dataset consisted of 151 samples from the macular or extramacular region of donor eye retinal pigmented epithelium. Data processing: to identify significant differentially expressed genes (SDEG) in data sets, we used the MultiExperiment Viewer (MeV) software. In cases where multiple genes probes have insisted on the same GeneID NCBI, we have used those with the highest variance (Table 3). Statistical analysis was performed using GEO2R, applying a Benjamini and Hochberg (False discovery rate) to adjust P values for multiple comparisons [66–68].

**Table 3:** Probes set list.

**N°**	**GSE**	**GPL**	**Probe set**	**Gene nomenclature**
1	44295	6883	ILMN_1692413	NMNAT1
			ILMN_1742968	NMNAT2
			ILMN_2153485	NMNAT3
			ILMN_1652207	COX4I1
			ILMN_2341363	ATP5A1
2	22138 27831 51880	570	223692_at	NMNAT1
			1562818_at	NMNAT2
			228090_at	NMNAT3
			227323_at	COX4I1
			1569891_at	ATP5A1
3	84976 73652	10558	ILMN_1692413	NMNAT1
			ILMN_1742968	NMNAT2
			ILMN_2153485	NMNAT3
			ILMN_1652207	COX4I1
			ILMN_2341363	ATP5A1
4	29801	4133	3719	NMNAT1
			25564	NMNAT2
			20338	NMNAT3
			39732	COX4I1
			37865	ATP5A1

### Statistical analysis

Data are shown as means ± standard error of the mean (SEM). For statistical analysis, Prism 7 software (GraphPad Software, USA) was used. Significant differences between groups were assessed using the one-way ANOVA test. Correlations were determined using Pearson’s ρ correlation. All tests were two-sided, and significance was determined at p < 0.05. The analysis of microarray data by Z-score transformation was used in order to allow the comparison of microarray data independent of the original hybridization intensities [34].

## Supplementary Material

Supplementary Figures
