# Supplementary material for: Loss of macroH2A1 decreases mitochondrial metabolism and reduces the aggressiveness of uveal melanoma cells
Source: Aging (Albany NY). 2020 May 12;12(10):9745–60. doi: 10.18632/aging.103241 (PMC7288915; doi:10.18632/aging.103241)
Supplement: Supplementary Figures [file aging-12-103241-s001..pdf]

SUPPLEMENTARY FIGURES

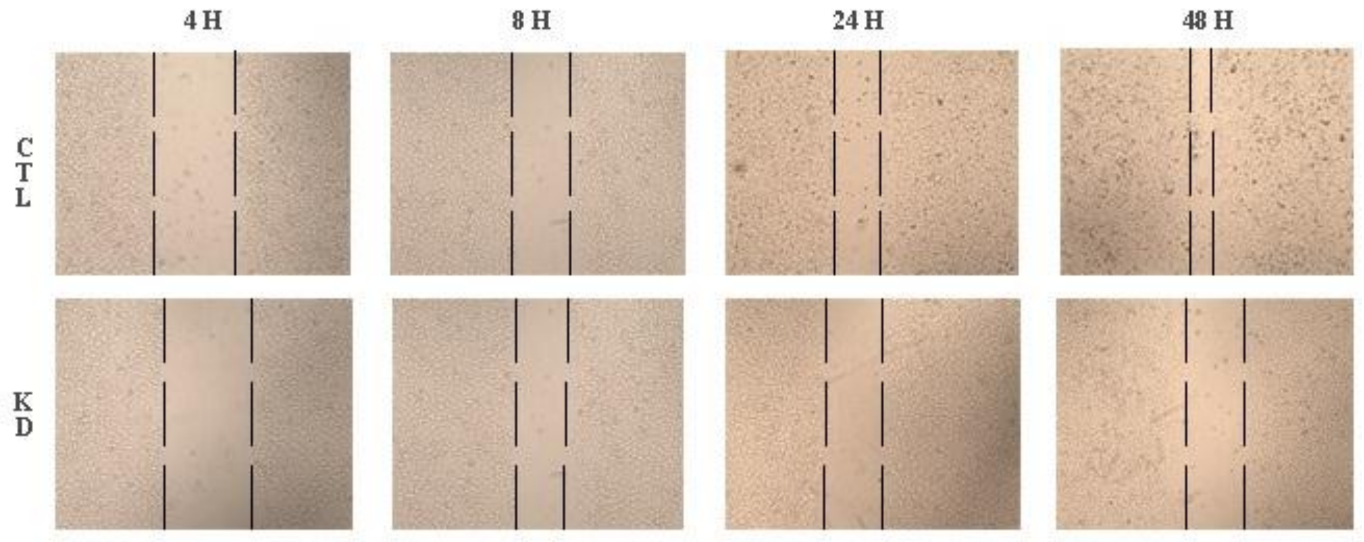

Supplementary Figure 1. Proliferation of 92.1 UM cells control (CTL) or knock-down (KD) for macroH2A1, in presence of serum. The number of the cells in the area of wound was measured for all the fields and time points (4h, 8h, 24h, 48h) using Image J.

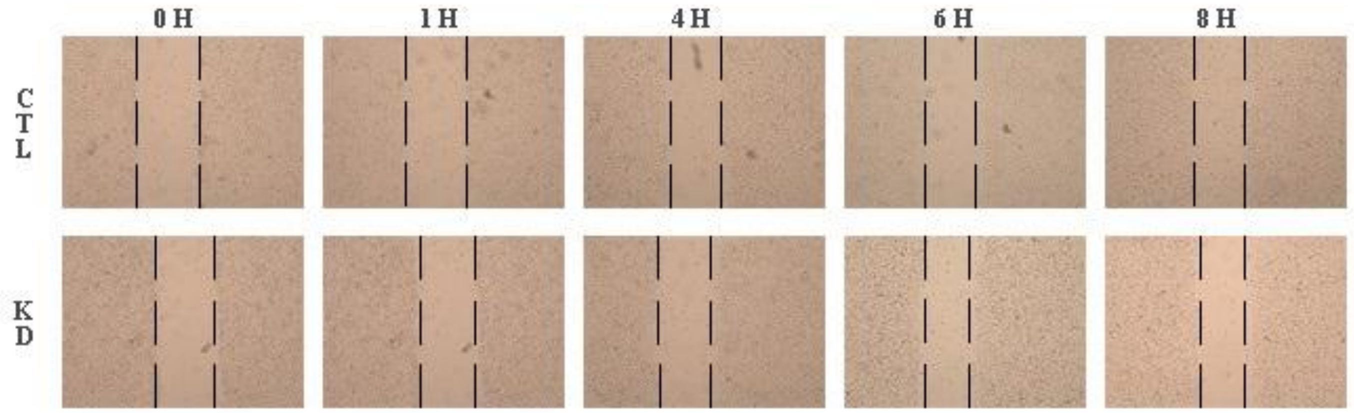

Supplementary Figure 2. Migration of 92.1 UM cells control (CTL) or knock-down (KD) for macroH2A1, with serum starvation. The number of the cells in the area of wound was measured for all the fields and time points (0h, 1h, 4h, 6h, 8h) using Image J.
